# Exploring the roles of cannot-link constraint in community detection via Multi-variance Mixed Gaussian Generative Model

**DOI:** 10.1371/journal.pone.0178029

**Published:** 2017-07-05

**Authors:** Liang Yang, Meng Ge, Di Jin, Dongxiao He, Huazhu Fu, Jing Wang, Xiaochun Cao

**Affiliations:** 1 School of Information Engineering, Tianjin University of Commerce, Tianjin, China; 2 State Key Laboratory of Information Security, Institute of Information Engineering, Chinese Academy of Sciences, Beijing, China; 3 School of Computer Software, Tianjin University, Tianjin, China; 4 School of Computer Science and Technology, Tianjin University, Tianjin, China; 5 Institute for Infocomm Research, Agency for Science, Technology and Research (A*STAR), Singapore, Singapore; 6 Faculty of Science and Technology, Bournemouth University, Bournemouth, United Kingdom; Beihang University, CHINA

## Abstract

Due to the demand for performance improvement and the existence of prior information, semi-supervised community detection with pairwise constraints becomes a hot topic. Most existing methods have been successfully encoding the must-link constraints, but neglect the opposite ones, i.e., the cannot-link constraints, which can force the exclusion between nodes. In this paper, we are interested in understanding the role of cannot-link constraints and effectively encoding pairwise constraints. Towards these goals, we define an integral generative process jointly considering the network topology, must-link and cannot-link constraints. We propose to characterize this process as a Multi-variance Mixed Gaussian Generative (MMGG) Model to address diverse degrees of confidences that exist in network topology and pairwise constraints and formulate it as a weighted nonnegative matrix factorization problem. The experiments on artificial and real-world networks not only illustrate the superiority of our proposed MMGG, but also, most importantly, reveal the roles of pairwise constraints. That is, though the must-link is more important than cannot-link when either of them is available, both must-link and cannot-link are equally important when both of them are available. To the best of our knowledge, this is the first work on discovering and exploring the importance of cannot-link constraints in semi-supervised community detection.

## Introduction

Networks have been ubiquitous in diverse fields, such as social networks, biological networks and technological networks, and attract many researchers to explore the sciences hid in the structures. Most of the networks in real life have a structure of community or modularity, which can embody the inhomogeneity of edge distribution. Communities, groups of nodes with high internal density, are of great importance and interesting in various domains. For instance, communities in scientist collaboration networks represent the same research topics, and in protein interaction networks nodes in the same community typically have the similar function. Therefore, identifying communities helps in exploring and understanding how the networks work. Albeit there is no universal definition of community structure, lots of algorithms have been proposed and achieved good performance [1–5]. But most of them identify communities using the network topology alone.

Recently, community detection using topology information combined with prior information becomes a hot topic, i.e. semi-supervised community detection [6–14]. This may be due to the following two reasons. On one hand, classical topology based community detection algorithms often cannot yield satisfactory results on networks where community structure is too complicated, such as overlapping or hierarchical properties. Recent researches on community detectability theoretically prove that any algorithms cannot correctly detect community structure if the difference between the number of intra and inter community edges is below a threshold [15]. On the other hand, the prior information is available in many real-world applications. Taking scientists collaboration network as an example, except the citation relationship as topology information, the title, key words and word frequency of the paper can be regarded as the prior knowledge. In addition, labels from the human are another source of the prior information. How to effectively and efficiently employ the prior information to enhance the performance of community detection is the key to semi-supervised community detection.

Prior knowledge utilized in existing methods can be categorized into two kinds, i.e., node label and pairwise label. Node label directly provides the relationship between a node and a community, i.e. indication of which community a node belongs to or must not belongs to [6,7]. Pairwise label builds the connections between two nodes, i.e. indicating whether two nodes belong to the same community [8–13]. If they belong to the same community, there exists a must-link between them. Whereas there exists a cannot-link. Compared with the node label, the pairwise label turns to be more readily available. Labeling whether two nodes belong to the same community would be much easier than labeling whether a node belongs to a certain community. For example, if we find that the keywords or tf-idf features of two web pages are very similar and they are under the same domain name, we can make use of this useful information to determine that they belong to the same community, i.e. must-link, even though they may not link with each other in network topology. Furthermore, the pairwise label can be used to represent the node label, but not vice versa. For example, if we know nodes A and B belong to community I and nodes C and D belong to community II, there exist must-links between nodes A and B and nodes C and D, and cannot-link between nodes A and C, nodes A and D, nodes B and C and node B and D. Therefore, we only consider employing pairwise label in semi-supervised community detection in this paper.

Usage of the pairwise label in most existing semi-supervised community detection algorithms can be divided into two categories, i.e., refining the network topology and characterizing node property, according to the role that pairwise supervised information acts. One group of methods first use the pairwise supervised information to refine the network topology, and then apply the existing unsupervised community detection algorithm to the refined networks [8–11]. By adding edges between must-link nodes and removing the edges between cannot-link nodes, Ma *et al*. modify the adjacency matrix of the network and adopt the symmetric nonnegative matrix factorization to detect community structure [8]. Zhang et al. extend this framework to other methods including modularity maximization model and Infomap algorithm [9]. Then Zhang et al. further extend this framework by adding a logical inference step to better utilize the supervised information [10]. This kind of methods often ignore the difference between the pairwise relationship in the network topology and pairwise constraint. In fact, however, pairwise constraint is much stronger than network topology. Specifically, the edge between two nodes only indicates there exist some relationships between them, but not implies they must belong to the same community, while the nodes with must-link must belong to the same community. And if there is not an edge between two nodes, they do not exist direct relationship, which does not imply they belong to different communities, while nodes with cannot-link really belong to the different communities. As reported by Zhang et al. the improvement from must-link constraints is much significant than that from cannot-link constraints [9–10]. This is because the edges in the network is sparse, thus adding equivalent must-link constraints could make intra-community edges much denser and improve the performance. The cannot-link constraints, however, cannot effectively cause the inter-community edges further sparse, thus the performance improvement is limited. In contrast, the other group of methods are based on the discriminative model which describes the node property in the detected communities [12,13]. Yang et al. propose a unified semi-supervised framework making the must-link (cannot-link) nodes have the similar (dissimilar) latent space representations which is the basis for classifying nodes into different communities [12]. Since this framework can distinguish the must-link constraints from the edge relationship, it achieves satisfactory result for must-link constraints. But it fails to encode cannot-link constraints for performance improvement, because the dissimilarity between the pair of cannot-link nodes cannot be properly defined.

In this paper, we aim to explore the effectiveness of cannot-link constraints on improving the community detection and further make the semi-supervised community detection with pairwise label more effective. To this end, we consider the generative model for semi-supervised community detection, which can effectively model the generation processes of network topology, must-link and cannot-link constraints together. We have the three findings on the nodes’ membership in the following.

1. A pair of nodes with must-link constraint must belong to the same community, i.e. if there is a must-link between two nodes, they belong to the same community with absolute confidence (probability). A pair of nodes with cannot-link constraint must not belong to the same community, i.e. if there is a cannot-link between two nodes, they have to belong to the different communities with very high confidence (probability).
2. If a pair of nodes belong to the same community, there exists an edge between them with a certain probability. If a pair of nodes belong to the different communities, there does not exist an edge between them with a certain confidence (probability).
3. The confidence (probability) in the second finding is much lower than that in the first one, since the pairwise constraints are much stronger than network topology.

Based on the first two findings, we assume that the network topology, must-link and cannot-link are generated based on the membership similarity of the pair of nodes together. Specifically, if **x**_*i*_ denotes the membership distribution of node *v*_*i*_, we let $x_{i}x_{j}^{T}$ represent the membership similarity between nodes *v*_*i*_ and *v*_*j*_. If we use *a*_*ij*_ ∈ {0, 1} as the indicator to denote whether there is an edge between nodes *v*_*i*_ and *v*_*j*_, we model the likelihood of the network topology as $N\left(x_{i}x_{j}^{T}|a_{ij},\;\sigma_{adj}\right)$ where *σ*_*adj*_ is the variance between the membership similarity and edge existence. Similarly, we model the likelihood of must-link and cannot-link constraints as $N\left(x_{i}x_{j}^{T}|1,\;\sigma_{ml}\right)$ and $N\left(x_{i}x_{j}^{T}|0,\;\sigma_{cl}\right)$, respectively. By combining the likelihoods of topology information, must-link constraint and cannot-link constraint together, we obtain the final likelihood of generating both the topology and constraint information. In addition, based on the aforementioned third finding we set *σ*_*adj*_ > *σ*_*ml*_ and *σ*_*adj*_ > *σ*_*cl*_, representing the higher confidence of constraint information over that of the topology information. Therefore, the membership indicator vector **x**_*i*_ *i* = 1,2 ⋯ *N* can be obtained by maximizing the likelihood of the generation of topology and constraints which is equivalent to minimizing the negative logarithmic function of the likelihood. This optimization can be solved by using the weighted symmetric nonnegative matrix factorization method which has the same complexity as the standard nonnegative matrix factorization.

The main contributions of this paper are two-fold: (1) We characterize semi-supervised community detection as a Multi-variance Mixed Gaussian Generative (MMGG) Model to address diverse degrees of confidences that exist in network topology and pairwise constraints and formulate it as a weighted nonnegative matrix factorization problem. (2) We reveal the roles of pairwise constraints, which is neglected by most researchers. That is, though the must-link is more important than cannot-link when either of them is available, both must-link and cannot-link are equally important when both of them are available.

## Results

To illustrate the effect of our proposed Multi-variance Mixed Gaussian Generative (MMGG) Model for semi-supervised community detection, we conduct experiments on two widely-used artificial benchmarks and six real-world networks ranging from social networks to technological networks. Here, we set *σ*_*adj*_ = 1 and vary both $1/\sigma_{ml}^{2}$ and $1/\sigma_{cl}^{2}$ from {2, 5, 10, 50, 100}. To demonstrate its superiority, we compare it with a baseline method recently proposed by Zhang et al [9]. This method refines the network topology using the pairwise supervised information, i.e., connects (disconnects) two nodes with must-link (cannot-link), and applies the symmetric nonnegative matrix factorization (SNMF) algorithm to the refined network to detect communities. We name this framework as ‘ModTop’ since it encodes supervised information by directly **mod**ify the **top**ology. The reasons why we take it as baseline are twofold. First, both ModTop and our proposed MMGG can make use of must-link and cannot-link constraints simultaneously. Second, they both take nonnegative matrix factorization as the key component to detect communities, which is fair for comparison. Normalized mutual information (NMI) is adapted to evaluate the performance improvement [16], since it is more informative than accuracy.

To fully explore and understand the effect of must-link and cannot-link constraint, we display the performance induced by must-link and cannot-link constraints respectively. In the ‘cannot-link’ subgraph, we use the following 5 methods for comparison:

**ModTop-CL** (blue dashed line with circle mark): encodes only cannot-link constraints via Zhang’s method,
**ModTop-MCL** (red dashed line with square mark): encodes both must-link and cannot-link constraints via Zhang’s method,
**MMGG-CL** (yellow dotted line with plus mark): encodes only cannot-link constraints via our proposed method,
**MMGG-CL(M)** (magenta dotted line with x-mark): encodes must-link constraints via Zhang’s method and encode cannot-link constraints via our proposed method,
**MMGG-MCL** (green solid line with star mark): encodes both must-link and cannot-link constraints via our proposed method.

The comparison of ModTop-CL and MMGG-CL is to illustrate the effectiveness of MMGG on encoding cannot-link constraint alone. The comparison of ModTop-MCL and MMGG-CL(M) is to show the improvement of MMGG on encoding cannot-link on the network topology which is modified by the must-link constraints. The comparison of MMGG-CL(M) and MMGG-MCL is to explore the different cannot-link constraints encoding effects caused by different must-link encoding methods. The difference between ModTop-MCL and MMGG-MCL is to illustrate the overall improvement of MMGG.

Similarly, in the ‘must-link’ subgraph, we use the following 5 methods for comparison:

**ModTop-ML** (blue dashed line with circle mark): encodes only must-link constraints via Zhang’s method,
**ModTop-MCL** (red dashed line with square mark): encodes both must-link and cannot-link constraints via Zhang’s method,
**MMGG-ML** (yellow dotted line with plus mark): encodes only must-link constraints via our proposed method,
**MMGG-ML(C)** (magenta dotted line with x-mark): encodes cannot-link constraints using Zhang’s method and encode must-link constraints via our proposed method,
**MMGG-MCL** (green solid line with star mark): encodes both must-link and cannot-link constraints via our proposed method.

The purposes of introducing these methods for comparison are similar with those in the cannot-link subgraph. To make the comparison clear, ModTop-MCL and MMGG-MCL are shown in both must-link graph and cannot-link graph for reference. The reason for this is that both them simultaneously encode the must-link and cannot-link and it is not appropriate to show them only in one sub-figure.

### Artificial benchmarks

Girvan-Newman (GN) benchmark [4] and Lancichinetti-Fortunato-Radicchi (LFR) benchmark [17] are two widely-used network generators which can randomly generate networks with specific parameters and known community structures. Network generated by GN network generator consists of four non-overlapping communities with the same size. Each community has 32 nodes each of which connects with 16 other nodes on average. Among these 16 edges, there are Z_*in*_ intra-community edges and Z_*out*_ inter-community edges, i.e., connecting Z_*in*_ nodes in the own community and Z_*out*_ nodes in the other communities and Z_*in*_ + Z_*out*_ = 16. These two parameters determine the clarity of the community structure and the detectability of the algorithms. Most of the methods, including nonnegative matrix factorization, modularity maximization and Infomap etc., achieve satisfactory results when Z_*out*_ ≤ 6, but significantly degrade as Z_*out*_ continue to increase.

**Fig 1:**
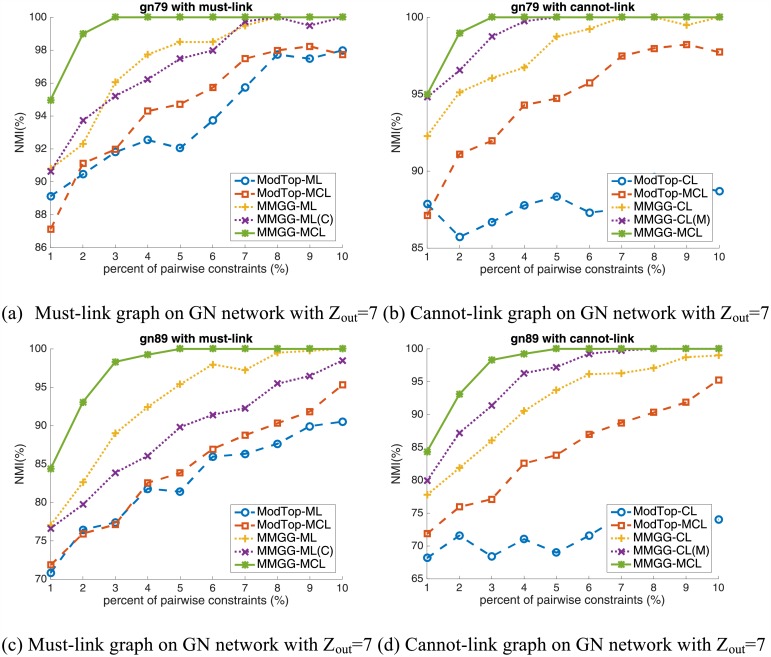
The performance on GN benchmark networks. The first (second) row is the results on networks with Z_*out*_ = 7 (Z_*out*_ = 8) and the first (second) column is the must-link graph (the cannot-link graph).

The performance of encoding pairwise constraints on GN network is shown in Fig 1, in which the first (second) row is the results on networks with Z_*out*_ = 7 (Z_*out*_ = 8) and the first (second) column is must-link graph (cannot-link graph).

From the results we find out the following three basic conclusions. 1) The performance of our proposed framework (MMGG) significantly outperforms that of the baseline method (ModTop) both on encoding the must-link constraints and on encoding cannot-link constraints. 2) The MMGG-MCL, i.e., embedding both the must-link and the cannot-link constraints using our framework, achieves the best performance which is much higher than other ModTop-related methods. For example, on GN networks with Z_*out*_ = 8, by encoding 3% constraints, ModTop-CL, ModTop-ML and ModTop-MCL achieve 69.3%, 77.3% and 77.4%, respectively. MMGG-CL, MMGG-CL(M), MMGG-ML and MMGG-ML(C) significantly increase to 86.0%, 91.3%, 88.5% and 83.8%, respectively. And the MMGG-MCL achieves 98.2%, which is at least 6.9% higher than MMGG-based methods with single constraints and at least 20.8% higher than TopMod-based methods. 3) On encoding single kind of constraints, the MMGG is more superior than TopMod. For example, the performance on encoding 5% percent must-link and cannot-link constraints by using TopMod on GN networks with Z_*out*_ = 7 are 92.7% 87.5% respectively. And those by using our proposed MMGG are both 98.7% which are 6% and 21.2% higher than the corresponding method based on TopMod.

**Fig 2:**
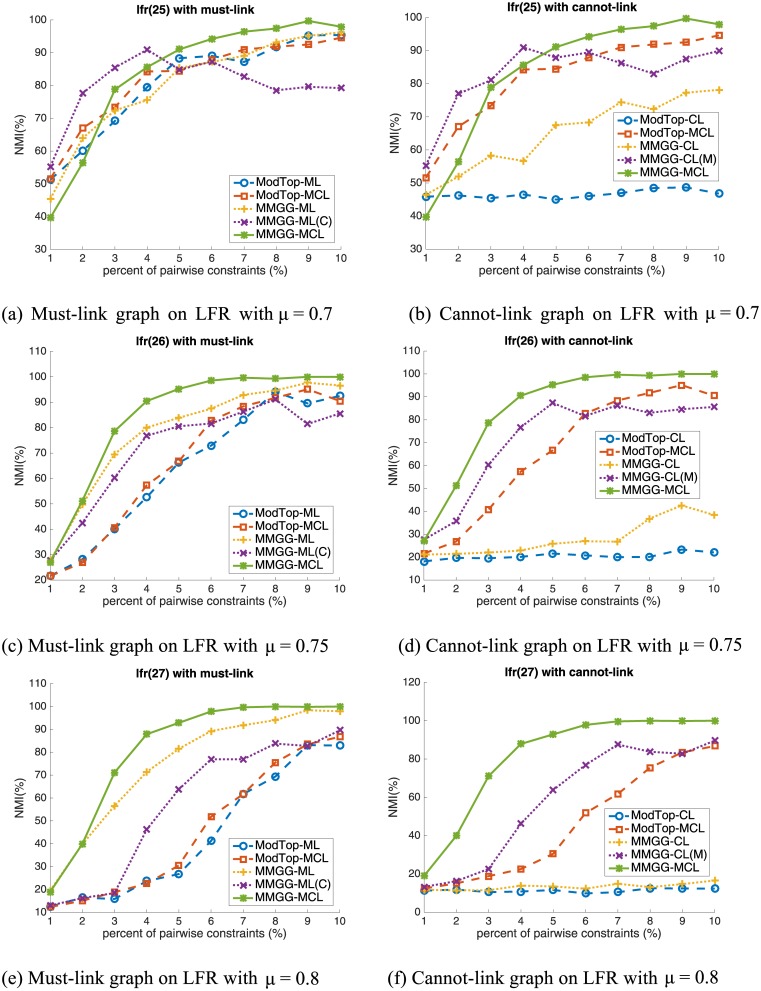
The performance on LFR benchmark networks. The first, second and third rows are the results with μ = 0.7, 0.75 and 0.8, respectively.

Compared with the GN benchmark, the LFR benchmark generator [17] is more complex and closer to the properties of real-world networks. Thus the community detection on LFR are more challenging and the results are more convincing. Different from the GN benchmark which fixes the node degree and community size, the distributions of node degree and community size obey power laws with parameters γ and β in LFR benchmark. Similar with the role of Z_*out*_ in GN benchmark, the fraction of inter-community edges (known as mixing parameter) μ can also be specific. Besides, we can further tune the minimum and maximum community size, and the number of nodes to make the generator more flexible. In this paper, we set the number of nodes to 1,000, the minimum community size to 10, the maximum community size to 5 times the minimum community size, the exponent of node degree distribution and community size distribution to 2 and 1, respectively as Lancichinetti et al [17]. do. Due to the important role of mixing parameter μ, we vary it from 0.7 to 0.8. The results are shown in Fig 2, where the first, second and third rows are the results with μ = 0.7, 0.75 and 0.8, respectively. The superiority of MMGG is more pronounced on vague networks, i.e. large mixing parameter μ on LFR network. This meets the purpose of our research and the scenario of semi-supervised community detection, i.e. improve the performance of community detection on networks where the community structure is vague and the performance is not satisfactory.

From these results in Fig 2, we can obtain the similar conclusions as in GN benchmark networks, expect the performance of MMGG-CL. Thus, we focus on the role analysis of must-link and cannot-link on performance improvement here. From the experimental results we draw the following conclusions. Firstly, as pointed by Zhang et al., the must-link constraint is more important than the cannot-link constraint on performance improvement. Taking networks with μ = 0.75 as an example, the performance of ModTop with 5% must-link constrains and 5% cannot-link constraints are 66.3% and 21.6% respectively. Though, the MMGG improves them to 83.9% and 25.8%, the performance of must-link is still much higher than that of cannot-link. Secondly, the performance can not be further improved and even degrades if cannot-link is not properly integrated with must-link. From the figures in the first column of Fig 2, we find that the performance of ModTop-ML and ModTop-MCL are very similar, which indicates that cannot-link constraints are meaningless in ModTop framework. But the performance of MMGG-ML is higher than MMGG-ML(C), which further illustrates that the superiority of MMGG on embedding cannot-link constraints. Thirdly, our proposed MMGG is more effective on encoding pairwise constraints, especially simultaneously encoding must-link and cannot-link constraints. On one hand, the performance on encoding must-link by MMGG (MMGG-ML) is much higher than that by ModTop (ModTop-ML). For example, on LFR networks with μ = 0.8, MMGG-ML achieves 83.9% while ModTop-ML only achieves 66.3%. On the other hand, based on the encoded must-link constraints by MMGG, MMGG can significantly improve the performance by additionally encoding cannot-link constraints. For example, with 5% cannot-link constraints, the performance is further improved from 83.9% to 95.3% on networks with μ = 0.75, and that is further improved from 81.5% to 92.9% on networks with μ = 0.8.

In summary, from the experiments on artificial networks, we obtain the following conclusions. 1) The must-link is more important for performance improvement than cannot-link if only one kind of pairwise constraint is available. 2) Both must-link and cannot-link are very important if both of them are available. The second conclusion is very different from that of Zhang et al. The reason why they obtain the flawed conclusion is that their encoding strategy is defective.

### Real-world networks

**Fig 3:**
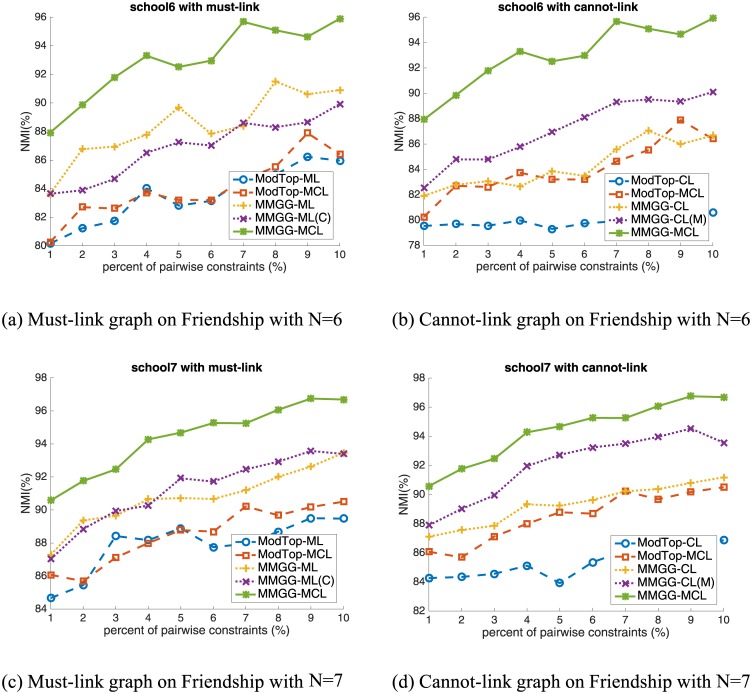
The performance on School Friendship Network. The first and second rows are the results with number of communities as 6 and 7, respectively.

**Fig 4:**
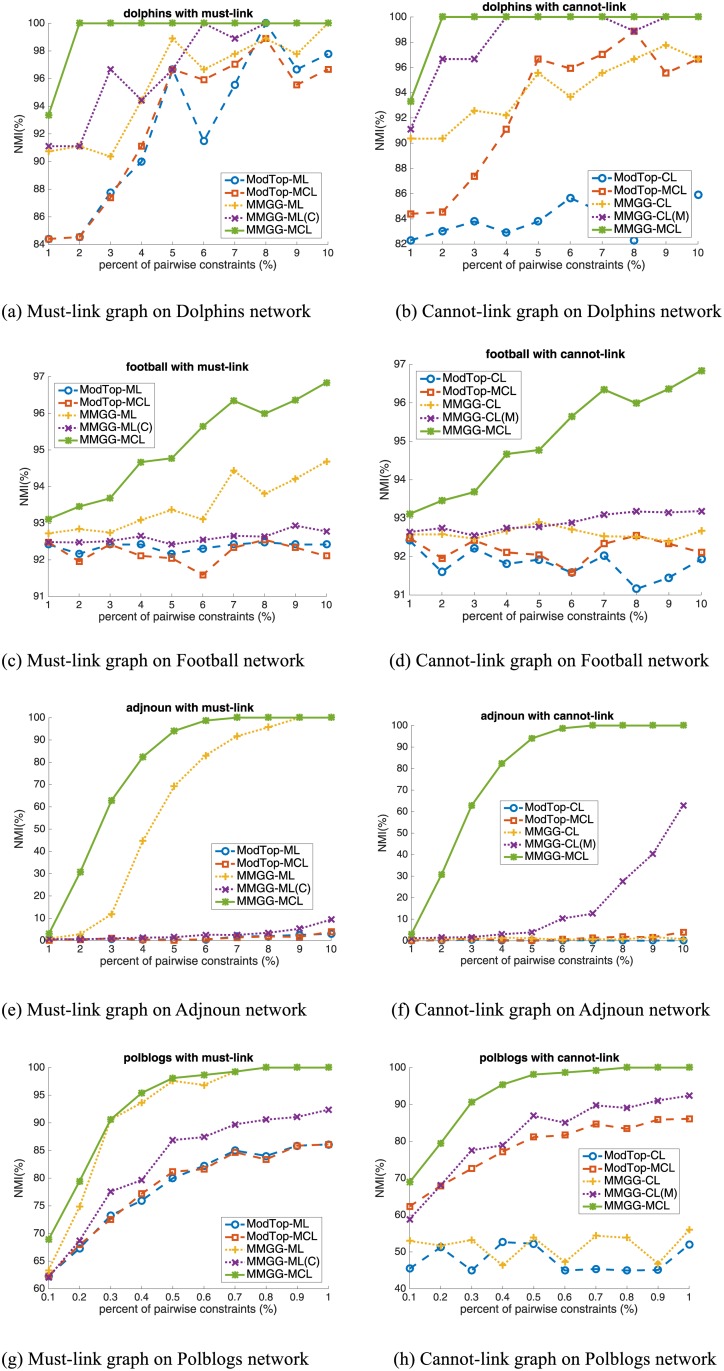
The performance on four real-world networks. 1^st^ row: Dolphins Network; 2^nd^ row: American College Football Network; 3^rd^ row: Adjnoun Network and 4^th^ row: Political Blogs Network.

**Fig 5:**
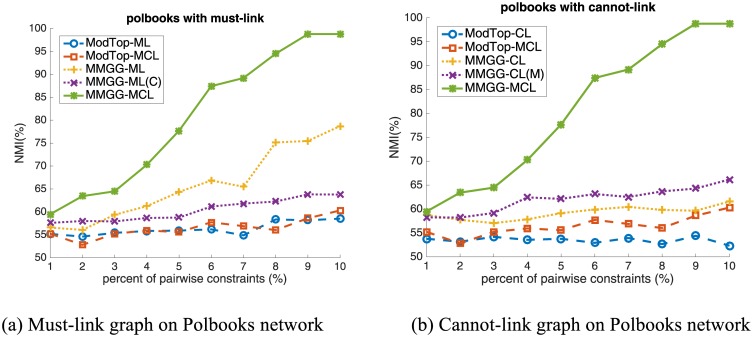
The performance on Political Books Network.

In this section, we verify our proposed MMGG on six real world networks with the same settings as on artificial networks. And the quantitative results are shown in Figs 3, 4 and 5.

**School Friendship Network** is one of the most popular social networks compiled by the National Longitudinal Study of Adolescent Health [18]. In the network, nodes represent the students from 6 different grades (7–12). Edges are the self-reporting friendship among them. The network can be divided into 6 communities according to students’ grade. Considering there are two sub-communities, i.e. white students and black students, in the community of grade 9, it is reasonable to divide the network into 7 communities. The results on School Friendship Network are shown in Fig 3. We can find that though the performance without constraints is acceptable, it can be further improved by encoding pairwise constraints. As shown in the left two sub-figures, the improvement from ModTop-ML to ModTop-MCL is very limited or even negligible (from 84.2% to 84.3% with 7% constraints), while that from MMGG-ML to MMGG-MCL is remarkable (from 89.0% to 95.1% with 7% constraints). This illustrates the important role of cannot-link constraints and indicates the effectiveness of our proposed MMGG on encoding pairwise constraints.

**Dolphins Social Network** is an undirected network reported by Lusseau [19]. In the network, two dolphins are connected if they are together more often than expected by chance. The 62 dolphins are classified into two communities, i.e. male dolphin community and female dolphin community. The results are shown in the first row of Fig 4. From the right sub-figure, we find the performance of encoding cannot-link constraints is significantly improved by MMGG (from blue dashed line to yellow dotted line). We also find with 1% percent of constraints encoded, the NMI of MMGG achieves 100%, which means all nodes are correctly classified. ModTop, however, needs 7% constraints to achieve 99%, which is 7 times that of MMGG. This fully shows the efficiency of MMGG on encoding pairwise constraints.

**American College Football Network** is an undirected network that reflects the relationship between American football teams among Division IA colleges during regular season Fall 2000 [4]. If two teams played against in that season, there is an edge between them in the network. The network is divided into 12 different communities according to their conferences. The results are in the second row of Fig 4. From a macro perspective, the performance improved by ModTop is limited (red dashed line), while that by MMGG is remarkable (green solid line). For example, by adding 7% constraints, ModTop improves from 92.1% to 92.4% (0.3% improved), while MMGG achieves 96.2% (4.1% improved). The performance improved by MMGG is about 14 times that by ModTop. From a micro perspective, in the left figure, the difference between MMGG-ML (yellow dotted line) and ModTop-ML (blue dashed line) shows the improvement of MMGG on must-link constraint, and the different between MMGG-MCL and MMGG-ML reflects the improvement of MMGG on cannot-link constraint. Both of them illustrate the high effectiveness of MMGG.

**Adjnoun Network** is an undirected network of common adjective and noun adjacencies for the novel "David Copperfield" by Charles Dickens [20]. Nodes represent the most commonly occurring adjectives and nouns in the book, and two words are linked if they occur in adjacent position in the book. The nodes are classified into “adjectives” community and “nouns” community. The results are presented in the third row of Fig 4. Since Adjnoun Network has anti-community structure, i.e., the inter-community edges are denser than the intra-community edges, most of the existing semi-supervised community detection methods, including ModTop, fail to achieve good results. Only the proposed MMGG-ML and MMGG-MCL effectively work on this network. To achieve 100% on NMI, MMGG-ML and MMGG-MCL only need 9% and 7% constraints. This case shows the superiority of MMGG on anti-community detection.

**Political Blogs Network**, which is compiled by Lada Adamic and Natalie Glance, is a directed network of hyperlinks between weblogs on US politics during the period of the 2004 presidential election [21]. The network topology is automatically extracted by a crawler, and the nodes are labeled manually labeled as “liberal” or “conservative”. The results are shown in the fourth row of Fig 4. From right figure, we can find that the performance improved by cannot-link is very limited on this network. However, since the MMGG is also more effective than ModTop on encoding must-link, the final performance of MMGG (green solid line) is much better than that of ModTop (red dashed line). By adding 0.5% constraints, ModTop improves the performance from 52.7% to 81.1%, while MMGG achieves 98.1%.

From experimental results on real world networks, we can draw similar conclusions as in artificial networks. In short, MMGG not only can effectively improve the performance on encoding single kind of pairwise constraints (the improvement from blue dashed line to the yellow dotted line in each plot), but also is superior on simultaneously encoding both of them (the improvement from red dashed line to green solid line).

### Case study

**Fig 6:**
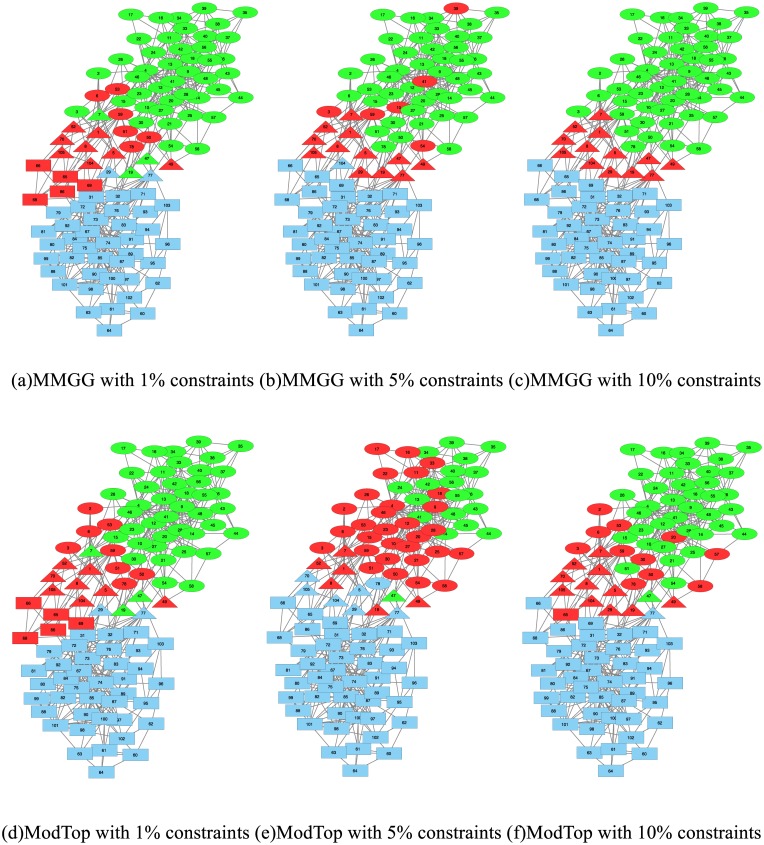
The detected communities by ModTop and MMGG on Political Books Network. The first row shows the results from MMGG, and the second shows those from ModTop.

Here we take Political Books Network [22] compiled by Valdis Krebs for case study. In the network, nodes represent books about US politics sold by the online bookseller Amazon.com, while two books are connected if they are frequently co-purchased by the same buyers. The network is divided into three communities, i.e., "liberal", "neutral" and “conservative", according to the views on US politics of the descriptions and the reviews of the books posted on Amazon. The performance of ModTop and MMGG are shown in Fig 5, which has the similar trend as on other real world networks. In order to make the results more intuitive, we visualize the results of ModTop (second row) and our proposed MMGG (first row) with adding 1%, 5% and 10% pairwise constraints (both must-link and cannot-link constraints) in Fig 6. The shape of nodes represents the ground-truth community which books belong to, i.e., “square” for “conservative” book, “circle” for “liberal” book and “triangle” for “neutral” book. The color of nodes represents the detected community by algorithms. We can find out that the performance of MMGG is still better than ModTop with 1% pairwise constraints, though neither of them can correctly detect the “neutral" book community since the boundary of community cannot be perfectly determined merely based on the network topology. Due to the high effectiveness of our proposed MMGG on encoding pairwise constraints, the boundary between “neutral" and “conservative” book community becomes clear with 5% of constraints. All nodes can be correctly classified by adding 10% constraints. The result of ModTop with 10% constraints is similar with that of MMGG with 5%, i.e. only one community boundary becomes clear, which further illustrates the effectiveness MMGG.

To further illustrate the scalability and complexity of our proposed MMGG, we test it on a larger social network, Facebook network for University of Pennsylvania from a date in Sept. 2005 [23]. This network contains 29,631 nodes, each of which represents a student. They are divided into 7 communities according to the year of enrollment. Without any prior information, the NMF-based method can obtain the community structure in 1,303 seconds, and the NMI of the result only achieves 22.1%. By adding 1% pairwise prior information, ModTop-MCL achieves 30.9% in 1,370 seconds, while our proposed MMGG-MCL achieves 64.8% in 1,981 seconds. The time spent on MMGG is about 1.5 times that spent on ModTop, while the performance improvement of MMGG is about 4.8 times that of the ModTop. Extra time spent on MMGG consists of two part. The first is the time used to compute element-wise product of weights matrix with other matrices. The second part mainly spends on the extra iterations for convergence. Since we amplify the impact of the reconstruction error from the pairwise prior constraints, we need more iterations to achieve the same convergence condition as in unsupervised version (the difference between successive iterations is less than 10^−3^).

### Parameter tuning

**Fig 7:**
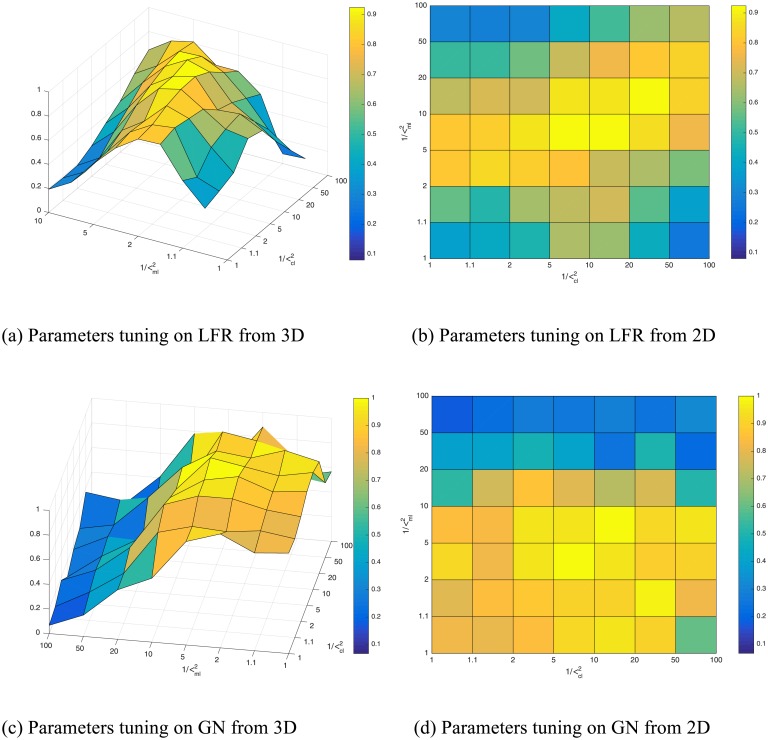
Parameters tuning. (a-b) Parameters tuning on LFR networks with μ = 0.8 and 5% pairwise constraints from 3D and 2D viewpoints. (c-d) Parameters tuning on GN networks with Z_*out*_ = 0.8 and 4% pairwise constraints from 3D and 2D viewpoints.

In this subsection, to make MMGG more practical, we exam the effect of the three variances, i.e., *σ*_*adj*_, *σ*_*ml*_ and *σ*_*ml*_, on performance improvement. To this end, we conduct experiments on LFR and GN benchmark networks. Since we use these three variances to model the confidences on generating the network topology and constraints, the ratios between them (*σ*_*adj*_ / *σ*_*ml*_ and *σ*_*adj*_ / *σ*_*cl*_), which reflect the differences of the confidences, are more important than the values. Therefore, we fix *σ*_*adj*_ = 1 and vary $1/\sigma_{ml}^{2}$ and $1/\sigma_{cl}^{2}$ from 1 to 100. Due to their similar trends, we only present the results on LFR networks with μ = 0.8 and 5% pairwise constraints in Fig 7(a) and 7(b) and those on GN networks with Z_*out*_ = 0.8 and 4% pairwise constraints in Fig 7(c) and 7(d). It shows that the performance is low if either $1/\sigma_{ml}^{2}$ or $1/\sigma_{cl}^{2}$ is small. And with the increases of $1/\sigma_{ml}^{2}$ and $1/\sigma_{cl}^{2}$, the performance is significantly improved. When $1/\sigma_{ml}^{2}$ and $1/\sigma_{cl}^{2}$ are in the vicinity of 5–10, the best performance is achieved. Therefore, we can set $\sigma_{adj}^{2}=1$, $\sigma_{ml}^{2}=0.2$ and $\sigma_{cl}^{2}=0.1$ in practice.

## Discussion

To understand the real roles of must-link and cannot-link constraints in semi-supervised community detection and improve the effectiveness of semi-supervised community detection on encoding pairwise constraints, we consider the generation process of the network topology, must-link and cannot-link constraints together. Due to the discovery that the network topology and pairwise constraints are generated with different degrees of confidence, we model this process as a Mixed Gaussian Model with Multi-variance. By maximizing the likelihood of the generative process on given network topology as well as the pairwise constraints, semi-supervised community detection can be solved via a weighted nonnegative matrix factorization method. The experiments on artificial and real-world networks reveal both the superiority of our proposed new method and the real roles of the pairwise constraints. On one hand, our proposed method can not only improve the performance on encoding single kind of pairwise constraints but also is superior on encoding must-link and cannot-link constraints together. On the other hand, and most importantly, although the must-link is more important for performance improvement than cannot-link when only one kind of pairwise constraint is available, but must-link and cannot-link are equally important to achieve better performance if they both are available. Though previous work also takes cannot-link constraints into consideration, most of them incorrectly conclude that the performance improved by cannot-link is limited and negligible due to their defective encoding strategy. To the best of our knowledge, this is the first work of discovering and exploring the important and real role of cannot-link constraints in semi-supervised community detection problem.

## Methods

A network can be modeled as a graph *G* = (*V*, *E*), where *V* = {*v*_1_, *v*_2_, ⋯, *v*_*N*_} is the set of *N* nodes and *E* = {(*v*_*i*_, *v*_*j*_)} is the set of *M* edges each of which connects two nodes, i.e., *v*_*i*_ and *v*_*j*_, in *V*. For convenience, we additionally define a set *NE* = {(*v*_*i*_, *v*_*j*_)} as the set of pairs of nodes which are not connected. The pairwise relationships in *E* and *NE* can also be equivalently represented as the adjacency matrix **A** = {*a*_*ij*_} ∈ {0,1}^*N*×*N*^ where *a*_*ij*_ = 1 if (*v*_*i*_, *v*_*j*_) ∈ *E* and *a*_*ij*_ = 0 if (*v*_*i*_, *v*_*j*_) ∈ *NE*. For simplicity, we assume *G* is an undirected and unweighted graph, and the adjacency matrix **A** is nonnegative symmetric binary matrix. Besides, we assume the number of communities *K* is known in advance. The must-link and cannot-link constraints are represented as *ML* = {(*v*_*i*_, *v*_*j*_)} and *CL* = {(*v*_*i*_, *v*_*j*_)}, respectively.

In the following, we consider the generative model of the semi-supervised community detection with pairwise constraint. The reason why the generative model is adopted is that it is more natural and convenient to describe the different strengths between the topology information and pairwise prior information. Specifically, we will use the variance of the Gaussian model to describe the confidence of information. We define the node membership matrix as $X=\left\{x_{ik}\right\}\in {\mathbb{R}}^{N\times K}$. Each row **x**_*i*_ denotes the probability distribution that node *v*_*i*_ belongs to different communities, and each element *x*_*ik*_ denotes the probability that node *v*_*i*_ belongs to community *k*. Thus *x*_*ik*_*x*_*jk*_ is the probability that both *v*_*i*_ and *v*_*j*_ belong to the community *k*, and $x_{i}x_{j}^{T}=\Sigma_{k=1}^{K}x_{ik}x_{jk}$ is the probability that they belong to the same community.

Firstly, we assume the probability that there exists a connection between *v*_*i*_ and *v*_*j*_ is determined by the probability that they belong to the same community, thus the likelihood of the existence of edges between then, i.e., *a*_*ij*_, is

$$\text{p}\left(x_{i}x_{j}^{T}|a_{ij}\right)=N\left(x_{i}x_{j}^{T}|a_{ij},\;\sigma_{adj}\right)=\frac{1}{\sqrt{2\pi \sigma_{adj}}}\text{exp}\left(-\frac{{\left(x_{i}x_{j}^{T}-a_{ij}\right)}^{2}}{2\sigma_{adj}^{2}}\right).$$

where N(x|μ, σ) denotes that the variable x conforms the Gaussian distribution with mean μ and variance *σ*. *σ*_*adj*_ is the parameter that measures the variance between the nodes’ membership similarity and the edge existence between them. Thus, the likelihood of generation of graph *G* is

$$\begin{array}{ll} \text{p}\left(X|A\right) & =\;\prod_{i,j=1}^{N}\text{p}\left(x_{i}x_{j}^{T}|a_{ij}\right)=\prod_{i,j=1}^{N}\frac{1}{\sqrt{2\pi \sigma_{adj}}}\text{exp}\left(-\frac{{\left(x_{i}x_{j}^{T}-a_{ij}\right)}^{2}}{2\sigma_{adj}^{2}}\right)\\ & =\prod_{\left(i,j\right)\in E}^{}\frac{1}{\sqrt{2\pi \sigma_{adj}}}\text{exp}\left(-\frac{{\left(x_{i}x_{j}^{T}-1\right)}^{2}}{2\sigma_{adj}^{2}}\right)\prod_{\left(i,j\right)\in NE}^{}\frac{1}{\sqrt{2\pi \sigma_{adj}}}\text{exp}\left(-\frac{{\left(x_{i}x_{j}^{T}-0\right)}^{2}}{2\sigma_{adj}^{2}}\right).\\ & =\text{p}\left(X|E\right)\text{p}\left(X|NE\right) \end{array}$$

Secondly, for each pair of nodes *v*_*i*_ and *v*_*j*_ in must-link constraint set *ML*, since they belong to the same community, their membership probability distribution **x**_*i*_ and **x**_*j*_ are very similar and $x_{i}x_{j}^{T}$ should be approximately 1. Therefore, we model the likelihood of generating the must-link constraints *ML* set as

$$\text{p}\left(X|ML\right)=\prod_{\left(i,j\right)\in ML}\text{ p}\left(x_{i}x_{j}^{T}|1\right)=\prod_{\left(i,j\right)\in ML}\frac{1}{\sqrt{2\pi \sigma_{ml}}}\text{exp}\left(-\frac{{\left(x_{i}x_{j}^{T}-1\right)}^{2}}{2\sigma_{ml}^{2}}\right).$$

Since the certainty that $x_{i}x_{j}^{T}\approx 1$ is very high, the variance *σ*_*ml*_ should be much smaller than *σ*_*adj*_. Similarly, the likelihood of generating the cannot-link constraint *CL* set can be modeled as

$$\text{p}\left(X|CL\right)=\prod_{\left(i,j\right)\in CL}\text{ p}\left(x_{i}x_{j}^{T}|0\right)=\prod_{\left(i,j\right)\in CL}\frac{1}{\sqrt{2\pi \sigma_{cl}}}\text{exp}\left(-\frac{{\left(x_{i}x_{j}^{T}-0\right)}^{2}}{2\sigma_{cl}^{2}}\right),$$

where *σ*_*cl*_ is also much smaller than *σ*_*adj*_. By combining the above analysis, the likelihood of generation the network topology and the pairwise constraint together is

p(X|A, ML, CL)= p(X|E)p(X|NE)p(X|ML)p(X|CL)

Since the must-link and cannot-link constraints should be mutually exclusive, thus there do not exist any pair of nodes which both belong to *ML* and *CL*. Thus, we can divide all pairs of nodes into the following six groups.

For (*i*, *j*) ∈ *E* ∩ *ML*, since *σ*_*ml*_ is much smaller than *σ*_*adj*_

$$\text{p}\left(\text{(}i,j)|A,\;ML,\;CL\right)\propto \text{exp}\left(-\frac{{\left(x_{i}x_{j}^{T}-1\right)}^{2}}{2\sigma_{adj}^{2}}-\frac{{\left(x_{i}x_{j}^{T}-1\right)}^{2}}{2\sigma_{ml}^{2}}\right)=\text{exp}\left(-\frac{\left(\sigma_{ml}^{2}+\sigma_{adj}^{2}\right){\left(x_{i}x_{j}^{T}-1\right)}^{2}}{2\sigma_{ml}^{2}\sigma_{adj}^{2}}\right)\approx \text{exp}\left(-\frac{{\left(x_{i}x_{j}^{T}-1\right)}^{2}}{2\sigma_{ml}^{2}}\right).$$

For (*i*, *j*) ∈ *NE* ∩ *CL*, since *σ*_*cl*_ is much smaller than *σ*_*adj*_

$$\text{p}\left((i,l)|A,\;ML,\;CL\right)\propto \text{exp}\left(-\frac{{\left(x_{i}x_{j}^{T}-0\right)}^{2}}{2\sigma_{adj}^{2}}-\frac{{\left(x_{i}x_{j}^{T}-0\right)}^{2}}{2\sigma_{cl}^{2}}\right)=\text{exp}\left(-\frac{\left(\sigma_{ml}^{2}+\sigma_{adj}^{2}\right){\left(x_{i}x_{j}^{T}-0\right)}^{2}}{2\sigma_{cl}^{2}\sigma_{adj}^{2}}\right)\approx \text{exp}\left(-\frac{{\left(x_{i}x_{j}^{T}-0\right)}^{2}}{2\sigma_{cl}^{2}}\right).$$

For (*i*, *j*) ∈ *E* \ (*ML* ∪ *CL*)

$$\text{p}\left(\text{(}i,j)|A,\;ML,\;CL\right)\propto \text{exp}\left(-\frac{{\left(x_{i}x_{j}^{T}-1\right)}^{2}}{2\sigma_{adj}^{2}}\right).$$

For (*i*, *j*) ∈ *NE* \ (*ML* ∪ *CL*)

$$\text{p}\left(\text{(}i,j)|A,\;ML,\;CL\right)\propto \text{exp}\left(-\frac{{\left(x_{i}x_{j}^{T}-0\right)}^{2}}{2\sigma_{adj}^{2}}\right).$$

For (*i*, *j*) ∈ *E* ∩ *CL*, since *σ*_*cl*_ is much smaller than *σ*_*adj*_

$$\begin{array}{c} \text{p}\left(\text{(}i,j)|A,\;ML,\;CL\right)\propto \text{exp}\left(-\frac{{\left(x_{i}x_{j}^{T}-1\right)}^{2}}{2\sigma_{adj}^{2}}-\frac{{\left(x_{i}x_{j}^{T}-0\right)}^{2}}{2\sigma_{cl}^{2}}\right)\\ =\text{exp}\left(-\frac{\left(\sigma_{adj}^{2}+\sigma_{cl}^{2}\right){\left(x_{i}x_{j}^{T}\right)}^{2}-\sigma_{cl}^{2}x_{i}x_{j}^{T}}{\sigma_{adj}^{2}\sigma_{cl}^{2}}\right)\;\approx \;\text{exp}\left(-\frac{{\left(x_{i}x_{j}^{T}-0\right)}^{2}}{2\sigma_{cl}^{2}}\right) \end{array}.$$

For (*i*, *j*) ∈ *NE* ∩ *ML*, since *σ*_*ml*_ is much smaller than *σ*_*adj*_

$$\begin{array}{c} \text{p}\left(\text{(}i,j)|A,\;ML,\;CL\right)\propto \text{exp}\left(-\frac{x_{i}x_{j}^{T}-0}{2\sigma_{adj}^{2}}-\frac{x_{i}x_{j}^{T}-1}{2\sigma_{ml}^{2}}\right)\\ =\text{exp}\left(\frac{\left(\sigma_{adj}^{2}+\sigma_{ml}^{2}\right){\left(x_{i}x_{j}^{T}\right)}^{2}-\sigma_{adj}^{2}x_{i}x_{j}^{T}}{2\sigma_{adj}^{2}\sigma_{ml}^{2}}\right)\;\approx \;\text{exp}\left(-\frac{{\left(x_{i}x_{j}^{T}-1\right)}^{2}}{2\sigma_{ml}^{2}}\right) \end{array}.$$

We summarize the means and variances for all the six groups in Table 1, and the final likelihood p(**X**|**A**, *ML*, *CL*) can be expressed as

$$\text{p}\left(X|A,\;ML,\;CL\right)=\prod_{i,j=1}^{N}\text{p}\left(\text{(}i,j)|A,\;ML,\;CL\right)=\;\prod_{i,j=1}^{N}\frac{1}{\sqrt{2\pi \sigma_{ij}}}\text{exp}\left(-\frac{{\left(x_{i}x_{j}^{T}-\mu_{ij}\right)}^{2}}{2\sigma_{ij}^{2}}\right).$$

**Table 1:** The means and variances for the generative model of all the six groups of node pairs.

Groups	Mean (μ_*ij*_)	Variance (*σ*_*ij*_)
(*i*, *j*) ∈ *E* ∩ *ML*	1	*σ*_*ml*_
(*i*, *j*) ∈ *E* ∩ *CL*	0	*σ*_*cl*_
(*i*, *j*) ∈ *NE* ∩ *ML*	1	*σ*_*ml*_
(*i*, *j*) ∈ *NE* ∩ *CL*	0	*σ*_*cl*_
(*i*, *j*) ∈ *E* \ (*ML* ∪ *CL*)	1	*σ*_*adj*_
(*i*, *j*) ∈ *NE* \ (*ML* ∪ *CL*)	0	*σ*_*adj*_

To inference the node membership matrix **X**, we can maximize the likelihood p(**X**|**A**, *ML*, *CL*). Since the monotonicity of logarithmic function, we can directly minimize

$$–\text{log}\left(\text{p}\left(X|A,\;ML,\;CL\right)\right)=\;\sum_{i,j=1}^{N}\frac{1}{2\sigma_{ij}^{2}}{\left(x_{i}x_{j}^{T}-\mu_{ij}\right)}^{2}+const_{ij}=\sum_{i,j=1}^{N}w_{ij}{\left(x_{i}x_{j}^{T}-o_{ij}\right)}^{2}+Const$$

Weight matrix $W=\left\{w_{ij}\right\}\in {\mathbb{R}}^{N\times N}$ where $w_{ij}=\frac{1}{2\sigma_{ij}^{2}}$ and new similarity matrix $O=\left\{o_{ij}\right\}\in {\mathbb{R}}^{N\times N}$ where *o*_*ij*_ = *μ*_*ij*_ as shown in Table 1. It is equivalent to a weighted symmetric nonnegative matrix factorization problem

$${\text{argmin}}_{X\geq 0}∥W⊙\left(XX^{T}-O\right){∥}_{F}^{2}.$$ (1)

where ⊙ means the element-wise product and ${\left\|X\right\|}_{F}=\sqrt{\sum_{i,j=1}^{N}x_{ij}^{2}}$ is the Frobenius norm of matrix **X**. Compared with the adjacency matrix **A** = {*a*_*ij*_} ∈ {0,1}^*N*×*N*^ where *a*_*ij*_ = 1 if (*v*_*i*_, *v*_*j*_) ∈ *E* and *a*_*ij*_ = 0 if (*v*_*i*_, *v*_*j*_) ∈ *NE*, **O** is equivalent to connecting the nodes with must-link constraints and disconnecting the nodes with cannot-link constraints based on **A**, which is the same as ModTop [9]. Therefore, ModTop can be regarded as a special case of our proposed MMGG in which each element of weight matrix **W** is 1. This means that ModTop ignores the difference between the topology information and pairwise constraints while our proposed MMGG takes it into consideration. MMGG amplifies the impact of the reconstruction error of the pairwise constraints by increasing the weights corresponding to the pairwise constraints. In the results section, we set the weights corresponding to pairwise constraints larger than 1 in MMGG-MCL, the those corresponding to must-link constraints larger than 1 in MMGG-ML(C) and those corresponding to cannot-link constraints larger than 1 in MMGG-CL(M).

To solve this constrained optimization problem, we construct the Lagrangian function as

$$\text{L}\left(X,\lambda \right)={\left\|W⊙\left(XX^{T}-O\right)\right\|}^{2}-\text{tr}\left(\lambda X^{T}\right),$$

where $\lambda =\left\{\lambda_{ij}\right\}\in {\mathbb{R}}^{N\times K}$ is the Lagrangian multiplier enforcing the nonnegative constraint on **X.** By letting the derivative of L(**X**, λ) with respect to **X** equal to 0, we get

$$\frac{\partial \text{L}\left(X,\lambda \right)}{\partial X}=4\left(W⊙\left(XX^{T}\right)⊙W^{T}\right)X-4\left(W⊙O⊙W^{T}\right)X-\lambda =0.$$

From the KKT condition $\lambda_{ij}X_{ij}^{4}=0$, we obtain

$${\left(\left(W⊙\left(XX^{T}\right)⊙W^{T}\right)X\right)}_{ij}X_{ij}^{4}-{\left(\left(W⊙O⊙W^{T}\right)X\right)}_{ij}X_{ij}^{4}=0.$$

The we get the following multiplication update rule

$$X_{ij}=X_{ij}{\left(\frac{{\left(\left(W⊙O⊙W^{T}\right)X\right)}_{ij}}{{\left(\left(W⊙\left(XX^{T}\right)⊙W^{T}\right)X\right)}_{ij}}\right)}^{\frac{1}{4}}.$$ (2)

For each pair of **W** and **O**, we randomly initialize **X** and iteratively update it using Eq (2) until it converges (the difference of losses between two consecutive iterations is less than 10^−3^) or reaches the maximum number of iterations (1000). This process is repeated for 20 times, and the **X** with the least loss is adopted as final result.

### Convergence analysis

**Definition 1:** $\text{G}\left(X,\hat{X}\right)$ is an auxiliary function for T(**X**) if the conditions $\text{G}\left(X,\hat{X}\right)\geq \text{T}\left(X\right),\;\text{G}\left(\hat{X},\hat{X}\right)=\text{T}\left(\hat{X}\right)$ are satisfied.

**Lemma 2:** If $\text{G}\left(X,\hat{X}\right)$ is an auxiliary function for T(**X**), the T(**X**) is nonincreasing under the update rule

$$\bar{X}={\text{arg min}}_{X}\;\text{G}\left(X,\hat{X}\right)$$

**Theorem 3.** The value of $\text{T}\left(X\right)={\left\|W⊙\left(XX^{T}-O\right)\right\|}_{F}^{2}$ is non-increasing under the update rule (2).

**Proof:** To prove this theorem we need to find an auxiliary function $\text{G}\left(X,\hat{X}\right)$ for $\text{T}\left(X\right)=\|W⊙\;\left(XX^{T}-O\right){\|}_{F}^{2}$**,** which satisfies

$$\text{G}\left(X,\hat{X}\right)\geq \text{T}\left(X\right),\;\text{G}\left(\hat{X},\hat{X}\right)=\text{T}\left(\hat{X}\right).$$

Minimizing T(**X**) is equivalent to minimizing

$$\text{S}\left(X\right)=\text{tr}\left(\left(W⊙\left(XX^{T}\right)\right)\left(\left(XX^{T}\right)⊙W^{T}\right)\right)-2\text{tr}\left(\left(W⊙\;\left(XX^{T}\right)\right)\left(O^{T}⊙W^{T}\right)\right).$$

We define the auxiliary function $\text{G}\left(X,\hat{X}\right)$ as

$$\text{G}\left(X,\hat{X}\right)=\sum_{i,j=1}^{N}\sum_{k=1}^{K}W_{ij}^{2}{\left(\hat{X}{\hat{X}}^{T}\right)}_{ij}{\hat{X}}_{ik}\frac{X_{jk}^{4}}{{\hat{X}}_{jk}^{3}}-2\sum_{i,j=1}^{N}\sum_{k=1}^{K}O_{ij}W_{ij}^{2}{\hat{X}}_{ik}{\hat{X}}_{jk}\left(1+\text{log}\frac{X_{ik}X_{jk}}{{\hat{X}}_{ik}{\hat{X}}_{jk}}\right)$$

It is easy to find $\text{G}\left(\hat{X},\hat{X}\right)=\text{S}\left(\hat{X}\right)$. We only need to prove $\text{G}\left(X,\hat{X}\right)\geq \text{S}\left(X\right)$. Since 1 + log *x* ≤ *x*

$$\begin{array}{l} -2\sum_{i,j=1}^{N}\sum_{k=1}^{K}O_{ij}W_{ij}^{2}{\hat{X}}_{ik}{\hat{X}}_{jk}\left(1+\text{log}\frac{X_{ik}X_{jk}}{{\hat{X}}_{ik}{\hat{X}}_{jk}}\right)\geq -2\sum_{i,j=1}^{N}\sum_{k=1}^{K}O_{ij}W_{ij}^{2}{\hat{X}}_{ik}{\hat{X}}_{jk}\frac{X_{ik}X_{jk}}{{\hat{X}}_{ik}{\hat{X}}_{jk}}=-2\sum_{i,j=1}^{N}\sum_{k=1}^{K}O_{ij}W_{ij}^{2}X_{ik}X_{jk}\\ =-2\text{tr}\left(\left(W⊙\left(XX^{T}\right)\right)\left(O^{T}⊙W^{T}\right)\right) \end{array}$$

Since *a*^4^ + *b*^4^ + *c*^4^ + *d*^4^ ≥ 4*abcd*, by setting $X_{jk}=\alpha_{jk}\;{\hat{X}}_{jk}$**,**

$$\begin{array}{l} \overset{N}{\underset{i,j=1}{\sum }}\overset{K}{\underset{k=1}{\sum }}W_{ij}^{2}{\left(\hat{X}{\hat{X}}^{T}\right)}_{ij}{\hat{X}}_{ik}\frac{X_{jk}^{4}}{{\hat{X}}_{jk}^{3}}=\overset{N}{\underset{i,j=1}{\sum }}\overset{K}{\underset{kh=1}{\sum }}W_{ij}^{2}{\hat{X}}_{ih}{\hat{X}}_{jh}{\hat{X}}_{ik}{\hat{X}}_{jk}x\alpha_{jk}^{4}\\ \geq \overset{N}{\underset{i,j=1}{\sum }}\overset{K}{\underset{kh=1}{\sum }}W_{ij}^{2}{\hat{X}}_{ih}{\hat{X}}_{jh}{\hat{X}}_{ik}{\hat{X}}_{jk}\alpha_{ih}\alpha_{ik}\alpha_{jh}\alpha_{jk}=\overset{N}{\underset{i,j=1}{\sum }}\overset{K}{\underset{kh=1}{\sum }}W_{ij}^{2}X_{ih}X_{jh}X_{ik}X_{jk}\\ =\text{tr}\left(W⊙\left(XX^{T}\right)\right)\left(\left(XX^{T}\right)⊙W^{T}\right) \end{array}$$

Therefore, we have $\text{G}\left(X,\hat{X}\right)\geq \text{S}\left(X\right)$. To make S(**X**) nonincreasing, we calculate the derivative of $\text{G}\left(X,\hat{X}\right)$ with respect to ***X***_***jk***_

$$\frac{\partial \text{G}\left(X,\hat{X}\right)}{\partial X_{jk}}\;=4\overset{N}{\underset{i,j=1}{\sum }}W_{ij}^{2}{\left(\hat{X}{\hat{X}}^{T}\right)}_{ij}{\hat{X}}_{ik}\frac{X_{jk}^{3}}{{\hat{X}}_{jk}^{3}}-4\overset{N}{\underset{i,j=1}{\sum }}O_{ij}W_{ij}^{2}{\hat{X}}_{ik}\frac{{\hat{X}}_{jk}}{{\hat{X}}_{jk}}\;=4{\left(\left(W⊙\left(XX^{T}\right)⊙W^{T}\right)X\right)}_{ij}\frac{X_{jk}^{3}}{{\hat{X}}_{jk}^{3}}-4{\left(\left(W⊙O⊙W^{T}\right)X\right)}_{ij}\frac{{\hat{X}}_{jk}}{X_{jk}}\;=0$$

Then we obtain the update role as

$$X_{ij}=X_{ij}{\left(\frac{{\left(\left(W⊙O⊙W^{T}\right)X\right)}_{ij}}{{\left(\left(W⊙\left(XX^{T}\right)⊙W^{T}\right)X\right)}_{ij}}\right)}^{\frac{1}{4}}.$$

### Complexity analysis

Here, we define the number of nodes, edges, communities and pairwise constraints as *N*, *M*, *K* and *P* respectively. The overall process of solving Multi-variance Mixed Gaussian Generative Model consists of the construction of weight matrix **W** and new similarity matrix **O** as shown in Eq (1) and solving the weighted nonnegative matrix factorization as shown in Eq (2). On one hand, from Table 1, we can obtain the new similarity matrix **O** from adjacency matrix **A** via assigning the elements corresponding to must-link as 1 and those corresponding to cannot-link as 0. Since we set *σ*_*adj*_ = 1, the weight matrix **W** can be obtained from a *N* × *N* matrix of ones by setting the elements corresponding to pairwise constraints as in Table 1. Thus, the process of weight and similarity matrix construction only needs *P* operations. On the other hand, the only differences between the Eq (2) and standard nonnegative matrix are the element-wise product of the new similarity and weight matrix and the element-wise product of **XX**^**T**^ and weight matrix. Since all elements in **W** are 1 except for *P* elements, the element-wise product only needs *P* multiple operations. Thus Eq (2) needs *P* + *N*^2^*K* operations. In a summary, the complexity of each iteration is O(*P* + *N*^2^*K*). O(*P* + *N*^2^*K*) can be further reduced to O(*N*^2^*K*) when *P* is given as a constant. Therefore, although MMGG effectively encodes the pairwise constraints, it still has the same complexity as the standard nonnegative matrix factorization and some semi-supervised community detection methods including Ma et al. [8] and ModTop [9]. This further illustrates the high efficiency of MMGG. Furthermore, since the main computation in our method is the matrix multiplication, and there are many parallel algorithms on it have been proposed, we can make use of parallel and distributed computing to make our framework applicable to more large-scale networks.
